# Extraction of high‐molecular‐weight DNA from *Streptococcus* spp. for nanopore sequencing in resource‐limited settings

**DOI:** 10.1002/mbo3.1432

**Published:** 2024-08-21

**Authors:** Suvra Das, JérÔme Delamare‐Deboutteville, Andrew C. Barnes, Oleksandra Rudenko

**Affiliations:** ^1^ School of the Environment and Centre for Marine Science The University of Queensland Saint Lucia Queensland Australia; ^2^ WorldFish Bayan Lepas Penang Malaysia

**Keywords:** high‐molecular‐weight DNA extraction, MinION rapid sequencing, nanopore, resource‐limited settings, *Streptococcus*, *Streptococcus iniae* plasmid

## Abstract

The long‐read sequencing platform MinION, developed by Oxford Nanopore Technologies, enables the sequencing of bacterial genomes in resource‐limited settings, such as field conditions or low‐ and middle‐income countries. For this purpose, protocols for extracting high‐molecular‐weight DNA using nonhazardous, inexpensive reagents and equipment are needed, and some methods have been developed for gram‐negative bacteria. However, we found that without modification, these protocols are unsuitable for gram‐positive *Streptococcus* spp., a major threat to fish farming and food security in low‐ and middle‐income countries. Multiple approaches were evaluated, and the most effective was an extraction method using lysozyme, sodium dodecyl sulfate, and proteinase K for lysis of bacterial cells and magnetic beads for DNA recovery. We optimized the method to consistently achieve sufficient yields of pure high‐molecular‐weight DNA with minimal reagents and time and developed a version of the protocol which can be performed without a centrifuge or electrical power. The suitability of the method was verified by MinION sequencing and assembly of 12 genomes of epidemiologically diverse fish‐pathogenic *Streptococcus iniae* and *Streptococcus agalactiae* isolates. The combination of effective high‐molecular‐weight DNA extraction and MinION sequencing enabled the discovery of a naturally occurring 15 kb low‐copy number mobilizable plasmid in *S. iniae*, which we name pSI1. We expect that our resource‐limited settings‐adapted protocol for high‐molecular‐weight DNA extraction could be implemented successfully for similarly recalcitrant‐to‐lysis gram‐positive bacteria, and it represents a method of choice for MinION‐based disease diagnostics in low‐ and middle‐income countries.

## Graphical abstract

A method for extracting high‐molecular‐weight DNA from *Streptococcus* spp., suitable for resource‐limited settings, was optimized. The extracted DNA was used for nanopore whole‐genome sequencing. This sequencing technique is valuable for the rapid diagnosis of diseases in warm water aquaculture, such as streptococcosis, under field conditions in low‐ and middle‐income countries. It provides near real‐time information on species, serotype, and the presence of plasmids. This was demonstrated by the discovery of a 15 kb low copy number mobilizable plasmid in *Streptococcus* iniae, the first reported in this species, which we named pSI1.

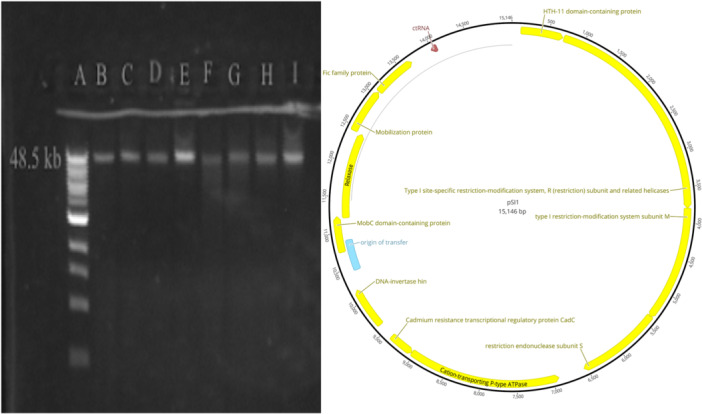

## 1 INTRODUCTION

The Oxford Nanopore Technologies (ONT) MinION sequencer is a low‐cost, pocket‐sized, portable long‐read sequencing device, which can be set up in resource‐limited settings (RLS) with low capital investment compared to other sequencing platforms (Leggett & Clark, 2017; Lu et al., 2016). For whole genome‐based bacterial disease diagnosis, serotyping, and epidemiological surveillance in low‐ and middle‐income countries (LMIC), it is a quick and convenient means of deriving comprehensive information on disease‐causing organisms. However, obtaining high‐molecular‐weight (HMW) DNA in sufficient quantity and purity is critical for optimal MinION sequencing. High amounts of extracted HMW DNA are required for sequencing using PCR‐free tagmentation‐based ONT kits offering rapid library preparation protocols optimal for RLS (Sauvage et al., 2023; Tyler et al., 2018). In a laboratory setting, DNA is typically extracted by methods that use either expensive or toxic reagents and special laboratory equipment. Although several alternative approaches appropriate to RLS applications were developed (Mason & Botella, 2020; Mirnejad et al., 2012), some for long‐read sequencing (Mayjonade et al., 2016), but only tested on gram‐negative bacteria. Therefore, it is essential to establish an equipment‐independent method for HMW DNA extraction that employs inexpensive and nontoxic reagents. This method should apply to a wide range of bacteria, including gram‐positive species that are recalcitrant to lysis, and to establish MinION‐based disease diagnostics in RLS.

DNA extraction methods comprise two basic stages: (1) lysis of cells and (2) separation of DNA from other cellular components such as proteins, lipids, and polysaccharides, as well as from the reagents used for cell lysis (Barbosa et al., 2016). In general, cell lysis by mechanical disruption such as bead beating is highly efficient, but it causes DNA fragmentation and should be avoided for long‐read MinION sequencing applications (Pinzauti et al., 2022; Yuan et al., 2012). Consequently, chemical treatments (e.g., sodium dodecyl sulfate [SDS]), enzymatic treatments (e.g., proteinase K), or their combination (e.g., SDS/proteinase K) are employed for the extraction of HMW DNA (Gill et al., 2016). SDS/proteinase K lysis is a nontoxic standard method used for high‐quality DNA extraction (Natarajan et al., 2016). However, considering the lower cost and wider availability, laundry detergent may be used as an alternative lysing agent in RLS (Mirnejad et al., 2012).

For DNA recovery/purification, one of the following basic methods can be employed: (1) phase separation, (2) column‐based separation, or (3) magnetic bead separation (Barbosa et al., 2016). The phase separation method includes harmful chemicals such as phenol and chloroform. Numerous commercial kits are available that avoid the use of toxic substances; however, they rely on costly silica columns for DNA purification. Both phase separation and commercial kits for DNA recovery utilize centrifugation, necessitating the use of a centrifuge and a power supply or battery. For RLS applications, a cellulose dipstick can be a simple, fast, cheap, and nontoxic alternative for DNA recovery from cell lysates without the need for laboratory equipment; however, the method's limitation is its low DNA yield (Mason & Botella, 2020). High yields of HMW DNA can also be recovered from cell lysates via capture on magnetic beads, a method that does not generate toxic waste and requires only a magnetic rack for equipment (Barbosa et al., 2016; Oberacker, Stepper, Bond, Höhn, et al., 2019).

*Streptococcus iniae* and *Streptococcus agalactiae* (Group B *Streptococcus*) are gram‐positive bacteria causing significant losses in finfish aquaculture in LMIC (Agnew & Barnes, 2007; Evans et al., 2009; Gill et al., 2016; Kawasaki et al., 2018; Shoemaker et al., 2001). Streptococcal strain diversity is high (Kawasaki et al., 2018), and pathogenicity to fish, antigenic composition (Deng et al., 2019; Heath et al., 2016; Kayansamruaj et al., 2019; Millard et al., 2012), and antibiotic sensitivity are strain dependent (Deng et al., 2019). Consequently, employing whole‐genome MinION sequencing of *Streptococcus* outbreak isolates appears to be the optimal strategy for disease management in LMIC aquaculture. This method facilitates rapid diagnosis of the causative agent and identification of treatment options, and offers the potential for disease prevention through surveillance of strain diversity, allowing for informed choice of strains for autogenous vaccine development (Barnes et al., 2022). However, due to the robust cell walls and high concentrations of carbohydrates present in thickly encapsulated *Streptococcus* spp., it may be difficult to release genomic DNA (Coleman et al., 1970; Şahin et al., 2016) without enzymatic pretreatment using lysozyme, mutanolysin, and/or lysostaphin (Coleman et al., 1970; Gill et al., 2016). Here, attempts were made to extract HMW DNA from *S. iniae* and *S. agalactiae* isolated from fish using inexpensive and nonhazardous DNA extraction approaches and their combinations. A low‐cost and efficient method was established and verified to be suitable for MinION sequencing.

## 2 MATERIALS AND METHODS

### 2.1 Establishment and optimization of the DNA extraction method

*S. iniae* strain QMA0248 (Alsheikh‐Hussain et al., 2022) was used to identify an effective DNA extraction protocol for fish‐pathogenic streptococci. Bacteria were recovered from −80°C stock on Tryptone Soya Agar supplemented with 5% sheep blood (TSA/SB) at 25°C for 24–48 h. Three to five colonies from the TSA/SB plate were picked and grown in Tryptone Soya Broth (TSB) overnight at 25°C. The density of bacterial culture was adjusted at 10^8^ CFU/mL (OD_600_ = 1.0 in Eppendorf BioPhotometer), and 1.5 mL culture was centrifuged to form a compact pellet.

DNA extraction followed five published protocols offering different cell lysis and recovery methods. These protocols were carried out as originally described or with modifications employing nontoxic and/or more cost‐effective and convenient reagents (Table 1). Each original and modified method was also performed with a lysozyme prelysis step (Ganz, 2006) using 16 mg/mL lysozyme in Tris‐EDTA (TE) buffer for 30 min at 37°C. Unless otherwise specified, SDS was used at 0.5% (v/v), and proteinase K was used at 0.1 mg/mL (as in our reference method described by Wilson [2001]). Laundry detergent treatment was performed using 1000, 500, 250, 125, 62.5, 30, 15, 7, 3, and 1 mg/L (weight by volume [w/v]) of OMO detergent powder (Unilever). The yield of recovered DNA and the amount of contaminating protein and RNA in samples were quantified with a Qubit 2.0 Fluorometer (Invitrogen). The integrity and molecular weight of DNA were assessed by electrophoresis (110 V for 35 min) on a 0.75% (w/v) agarose gel.

**Table 1:** Combinations of methods for cell lysis and DNA recovery used for DNA extraction (References are provided for combinations applied in the published protocols).

Method#	Cell lysis^a^	DNA recovery	Hazard/toxicity
1.	SDS/proteinase K/CTAB	Phenol‐chloroform	Toxic lysis (CTAB) and toxic recovery (phenol, chloroform) (Wilson, 2001)
	OMO/proteinase K		Nontoxic lysis and toxic recovery (phenol, chloroform)
2.	OMO	Ethanol	Nontoxic lysis and recovery (Mirnejad et al., 2012)
	SDS/proteinase K		Nontoxic lysis and recovery
3.	SDS/proteinase K	Cellulose dipstick	Nontoxic lysis and recovery (Mason & Botella, 2020)
	OMO/proteinase K		Nontoxic lysis and recovery
4.	GITC	Isopropanol/magnetic beads	Toxic lysis (GITC) and nontoxic recovery (Oberacker, Stepper, Bond, Höhn, et al., 2019)
	SDS/proteinase K		Nontoxic lysis and nontoxic recovery
	OMO/proteinase K		Nontoxic lysis and nontoxic recovery
5.	SDS	PEG8000/magnetic beads	Nontoxic lysis and recovery (Mayjonade et al., 2016)
	OMO		Nontoxic lysis and recovery

Abbreviations: CTAB, cetyltrimethylammonium bromide; GITC, guanidinium isothiocyanate; OMO, OMO detergent laundry powder; PEG8000, polyethylene glycol 8000; SDS, sodium dodecyl sulfate.

^a^ In all cases performed with and without lysozyme pretreatment.

Method 1: SDS‐proteinase K‐CTAB lysis/phenol‐chloroform recovery

The phase separation method utilizing SDS/proteinase K/CTAB, as described by Wilson (2001), has been routinely employed in our laboratory to extract DNA intended for MinION sequencing (Wilson, 2001). Despite its effectiveness, this method uses toxic reagents. Therefore, here we used this method as a reference to evaluate the efficiency of alternative extraction methods suitable for RLS. In addition, a laundry detergent lysis method (Mirnejad et al., 2012) was used with this protocol, substituting SDS with OMO detergent powder. OMO is a globally available laundry detergent manufactured by Unilever and sold under the brand names, OMO, Persil, Skip, and Surf, depending upon location.

Method 2: Laundry detergent powder lysis/ethanol recovery

This method uses laundry detergent powder for cell lysis and ethanol precipitation to recover the DNA (Mirnejad et al., 2012). It was applied without modification (using 1–1000 mg/L OMO powder) and with an alternative lysis step using SDS/proteinase K lysis instead of the laundry detergent lysis.

Method 3: SDS‐proteinase K lysis/cellulose dipstick recovery

A cellulose dipstick can be used to capture DNA from various kinds of lysates (Mason & Botella, 2020)*.* This method was used to recover streptococcal DNA from both SDS/proteinase K lysates and OMO powder/proteinase K lysates.

Method 4: GITC lysis/magnetic bead recovery

This method employs toxic guanidinium‐isothiocyanate (GITC) based lysis buffer, isopropanol to precipitate the DNA, and 20 μL of in‐house synthesized magnetic beads to capture the precipitate (Oberacker, Stepper, Bond, Höhn, et al., 2019). We performed lysis as described and using two nontoxic lysis approaches: SDS/proteinase K and OMO/proteinase K treatments. DNA was recovered using 20 μL of the commercial AMPure XP beads (Beckman Coulter).

Method 5: SDS lysis/magnetic bead recovery

This method was developed for long‐read sequencing applications. It employs a multireagent buffer containing 1.25% SDS for lysis, potassium acetate and centrifugation for removal of protein and polysaccharide contaminants, PEG8000 for DNA precipitation, and 20 μL Sera‐Mag SpeedBeads magnetic beads washed four times in multicomponent buffer for DNA capture (Mayjonade et al., 2016). Initially, the protocol was performed exactly as described except for using AMPure XP beads (without the recommended washing) and using two alternative lysis treatments: 1.25% SDS/0.74 mg/mL proteinase K and 1 mg/L OMO/0.74 mg/mL proteinase K. As with other methods listed above, each lysis combination was carried out with or without lysozyme treatment. The incubation with potassium acetate was performed at ambient temperature or omitted.

Subsequently, the protocol was performed using lysozyme/SDS/proteinase K and lysozyme/OMO/proteinase K treatments, applied to bacterial pellets from 1.5, 3, and 4 mL of input of broth culture, with removal of contaminants precipitated by potassium acetate performed using either centrifugation (as described) or 0.45 µm filter‐syringe filtration.

Finally, lysates from 1.5 mL culture pellets treated with lysozyme/0.5% SDS; 0.3 or 0.5 mg/mL proteinase K; and 0.16, 0.4, or 0.8 mg/mL RNaseA, were used to purify DNA with either centrifugation or syringe‐filtration (0.22 µm filter) to remove the contaminants, and either PEG8000 buffer (2 g of PEG8000 and 1.75 g of sodium chloride in 10 mL nuclease‐free water) or isopropanol to precipitate the DNA.

### 2.2 Verification of the DNA extraction method

After setting up an efficient final protocol using *S. iniae* strain QMA0248 (Section 2.1), the method was applied to 12 diverse streptococcal strains comprising seven isolates of *S. iniae* and five isolates of *S. agalactiae* (Table 2). Isolates were chosen to represent phylogenetically, ecologically, and phenotypically different groups (Irion et al., 2021; Kawasaki et al., 2018; Rudenko et al., 2020).

**Table 2:** *Streptococcus* spp. strains used for DNA extraction and sequencing (Irion et al., 2021; Kawasaki et al., 2018; Rudenko et al., 2020).

SN	Species and strain	Host	Year	Geographic location	Sequence type (ST)
1.	*S. iniae* QMA0084	Flying fox (*Epalzeorhynchos kalopterus*)	2001	Australia	ST‐6
2.	*S. iniae* QMA0139	Tilapia (*Oreochromis* sp.)	1996	Canada	ST‐9
3.	*S. iniae* QMA0177	Barramundi (*Lates calcarifer*)	2006	Australia	ST‐8
4.	*S. iniae* QMA0186	Rainbow trout (*Oncorhynchus mykiss*)	2000	Israel	ST‐11
5.	*S. iniae* QMA0249	Barramundi (*L. calcarifer*)	2009	Australia	ST‐10
6.	*S. iniae* QMA0445	Tilapia (*Oreochromis* sp.)	1998	USA	ST‐4
7.	*S. iniae* QMA0462	Clown loach (*Chromobotia macracanthus*)	2001	USA	ST‐6
8.	*S. agalactiae* QMA0274	Mullet (*Ellochelon vaigiensis;* renamed from *Liza vaigiensis*)	2009	Australia	ST‐261
9.	*S. agalactiae* QMA0321	Estuary stingray (*Hemitrygon fluviorum*; renamed from *Dasyatis fluviorum*)	2010	Australia	ST‐261
10.	*S. agalactiae* QMA0496	Tilapia (*Oreochromis niloticus*)	2015	Honduras	ST‐260
11.	*S. agalactiae* QMA0522	Estuary stingray (*H. fluviorum;* renamed from *D. fluviorum*)	2018	Australia	ST‐261
12.	*S. agalactiae* QMA0539	Tilapia (*O. niloticus*)	2017	Vietnam	ST‐283

Abbreviation: ST, multilocus sequence type.

The suitability of the extracted DNA for MinION sequencing was ascertained as follows: a 12‐sample barcoded library was prepared using the Rapid Barcoding Sequencing Kit (SQK‐RBK004) following the manufacturer's instructions, loaded and sequenced onto the MinION flow cell FLO‐MIN106D (R9.4.1), and mounted on the MinION Mk1B device. Base calling was performed post‐run using the Guppy module v. 5.0.16 under High Accuracy mode and quality threshold >7. Genomes were assembled with Flye v 2.8.3 (Kolmogorov et al., 2019), assembly graphs visualized using Bandage v 0.8.1 (Wick et al., 2015), and annotation performed with Prokka v 1.12 (Seemann, 2014). Annotated contigs were visualized and analyzed in Geneious Prime 2023.0.4 (https://www.geneious.com).

For comparative purposes of the genome assembly statistics and to verify the presence of the plasmid in *S. iniae* QMA0139, DNA was also extracted from this strain using the reference lysozyme + CTAB method, which was sequenced and assembled as above. Genome assemblies were annotated with PGAP (Tatusova et al., 2016) and deposited at NCBI Genome.

### 2.3 Extraction and size estimation of natural *S. iniae* plasmid (named here pSI1)

**Figure A1:**
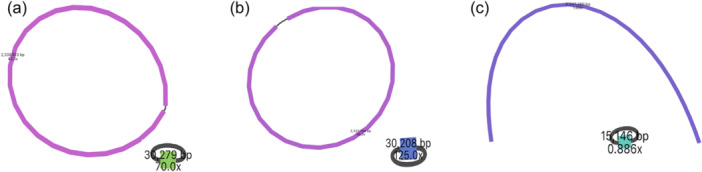
Assemblies of *Streptococcus iniae* QMA0139 genome visualized in Bandage: (a) DNA extracted using resource‐limited settings method, Flye assembly of Nanopore reads (CP159896–CP159897); (b) DNA extracted using lysozyme‐cetyltrimethylammonium bromide reference method, Flye assembly of Nanopore reads (CP159894–CP159895); (c) Hybrid assembly of Illumina reads (SRR7151914) and Nanopore reads from this study performed by Unicycler (CP158020–CP158021).

**Figure B1:**
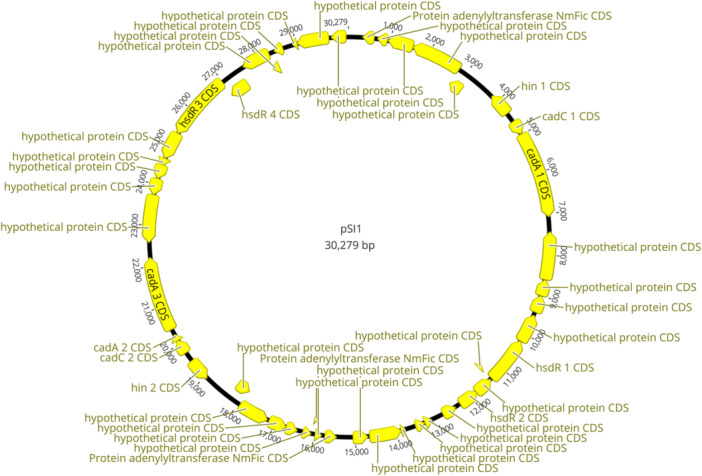
pSI1 plasmid dimer contig (CP159895; misassembly) from genome assembly of *Streptococcus iniae* QMA0139 generated by Flye, annotated with PROKKA, and visualized in Geneious Prime.

**Figure C1:**
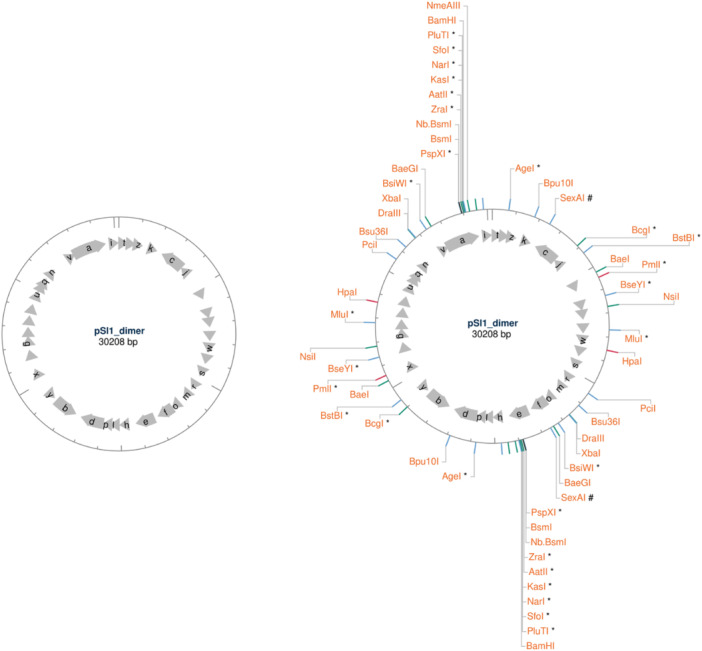
Restriction cutting sites identified by NEB Cutter v3.0 in 30 kb dimeric sequence of pSI1 (CP159895): left – 1‐cutters; right – 2‐cutters.

The putative 30 kb plasmid dimer discovered in genome assemblies of *S. iniae* QMA0139 performed with Flye (both from DNA extracted by RLS‐protocol and lysozyme + CTAB protocol; Appendix Figures A1a,b and B1) was extracted by alkaline lysis. Overnight QMA0139 culture in 300 mL TSB was pelleted and treated with 20 mg/mL lysozyme in 20 mL of PBS for 1 h at 37°C. Lysozyme‐treated culture was pelleted by centrifugation at 2000×*g*, resuspended in 10 mL of R1 buffer from PureLink HiPure Plasmid Midiprep Kit (Invitrogen), and processed according to the manufacturer's instruction. One microgram of extracted pSI1 was visualized on 0.5% agarose gel undigested, digested with putative 2‐site restriction cutter NsiI (NEB), and 4‐site cutter (as predicted for the dimeric sequence by NEB cutter v 3.0; Appendix Figure C1). The monomeric form of the plasmid suggested by gel migration of the undigested sample was further verified by mapping the reads onto 30 kb contig. To obtain the assembly of ~15 kb plasmid monomer, a short‐read‐first hybrid assembly was carried out with Unicycler 0.4.8 (Wick et al., 2017) using Illumina reads available at NCBI (SRR7151914) and long reads from this study (Appendix Figure A1c). pSI1 contig from this assembly was annotated with Bakta v1.9.1 (Schwengers et al., 2021).

## 3 RESULTS

Initially, the effective and reproducible HMW DNA extraction approach for fish‐pathogenic streptococci was chosen after testing five published protocols as originally described or with RLS‐appropriate modifications (Section 3.1). The established method was further optimized (Section 3.2) and verified by sequencing of phylogenetically and ecologically diverse streptococcal isolates (Section 3.3).

### 3.1 Selection of the extraction method

The quantity of recovered DNA and amount of protein contamination obtained from the extracted DNA samples using five published protocols and their modified versions are listed in Table 3.

**Table 3:** Testing different DNA extraction methods with various lysis and DNA recovery/purification combinations.

SN	Lysis	DNA recovery/purification	DNA (ng/μL)	Protein (ng/μL)
1.	SDS^1^/proteinase K^1^/CTAB (Wilson, 2001)	Phenol‐chloroform (Wilson, 2001)	<0.1	<1.0
	Lysozyme/SDS^1^/proteinase K^1^/CTAB		133	<1.0
	OMO^1^/proteinase K^1^		<0.1	110
	Lysozyme/OMO^1^/proteinase K^1^		<0.1	100
2.	OMO^1^ (Mirnejad et al., 2012)	Ethanol (Mirnejad et al., 2012)	<0.1	ND
	Lysozyme/OMO^1^/proteinase K^1^		<0.1	ND
	SDS^1^/proteinase K^1^		<0.1	ND
	Lysozyme/SDS^1^/proteinase K^1^		<0.1	ND
3.	SDS^1^/proteinase K^1^ (Mason & Botella, 2020)	Cellulose dipstick (Mason & Botella, 2020)	<0.1	ND
	Lysozyme/SDS^1^/proteinase K^1^		<0.1	ND
	OMO^1^/proteinase K^1^		<0.1	ND
	Lysozyme/OMO^1^/proteinase K^1^		<0.1	ND
4.	GITC (Oberacker, Stepper, Bond, Höhn, et al., 2019)	Isopropanol/Magnetic beads (Oberacker, Stepper, Bond, Höhn, et al., 2019)	<0.1	<1.0
	Lysozyme/GITC		<0.1	<1.0
	SDS^1^/proteinase K^1^		<0.1	<1.0
	Lysozyme/SDS^1^/proteinase K^1^		5.80	<1.0
	OMO^1^/proteinase K^1^		<0.1	<1.0
	Lysozyme/OMO (30 mg/L)/proteinase K^1^		72	4080
	Lysozyme/OMO (15 mg/L)/proteinase K^1^		71.3	2960
	Lysozyme/OMO (7 mg/L)/proteinase K^1^		74.2	2980
	Lysozyme/OMO (3 mg/L)/proteinase K^1^		45.6	1270
	Lysozyme/OMO (1 mg/L)/proteinase K^1^		63.0	1130
5.	SDS^2^ (Mayjonade et al., 2016)	Potassium acetate/PEG8000/Magnetic beads (Mayjonade et al., 2016)	<0.1	100
	Lysozyme/SDS^2^/proteinase K^2^	PEG8000/Magnetic beads	7.42	361.5
	SDS^2^/proteinase K^2^		<0.1	110
	Lysozyme/OMO^2^/proteinase K^2^		4.3	557.5
	OMO^2^/proteinase K^2^		<0.1	212
	Lysozyme/SDS^2^/proteinase K^2^	Potassium acetate/PEG8000/Magnetic beads (Mayjonade et al., 2016)	43.5	110
	SDS^2^/proteinase K^2^		4.43	120
	Lysozyme/OMO^2^/proteinase K^2^		3.52	134
	OMO^2^/proteinase K^2^		2.42	126

*Note*: Lysozyme – 0.16 mg/mL, SDS^1^ = 0.5%, SDS^2^ = 1.25%, OMO^1^ = concentrations tested were 1000, 500, 250, 125, 62.5, 30, 15, 7, 3, and 1 mg/L (w/v) OMO^2^ = concentration tested was 1 mg/L (w/v), proteinase K^1^ = 0.1 mg/mL, proteinase K^2^ = 0.74 mg/mL.

Abbreviations: CTAB, cetyltrimethylammonium bromide; GITC, guanidinium isothiocyanate; ND, not detected; OMO, OMO detergent laundry powder; PEG8000, polyethylene glycol 8000; SDS, sodium dodecyl sulfate.

The method employing SDS/proteinase K/CTAB for lysis and phenol‐chloroform DNA recovery (Wilson, 2001) failed to extract DNA when carried out as originally described. However, the concentration/amount of DNA extracted from *S. iniae* QMA0248 was more than enough for MinION sequencing (>100 ng/μL) when treatment with 16 mg/mL lysozyme was performed before SDS/proteinase K/CTAB lysis. Substitution of SDS lysis with laundry powder OMO lysis did not allow the recovery of significant amounts of DNA, even when the lysozyme treatment step was included.

Laundry powder (OMO) lysis followed by DNA recovery with ethanol (Mirnejad et al., 2012), was unsuccessful in extracting DNA, even, when samples were pretreated with lysozyme. This was also true for ethanol recovery from OMO/proteinase K and SDS/proteinase K lysates. Likewise, no detectable DNA was recovered from the above lysates using the cellulose dipstick method (Mason & Botella, 2020).

The approach employing GITC‐based buffer lysis followed by DNA recovery using magnetic beads (Oberacker, Stepper, Bond, Höhn, et al., 2019) was also inefficient in DNA extraction when carried out as originally described. Substitution of the multicomponent GITC‐based lysis buffer by lysozyme/SDS/proteinase K treatment permitted recovery of DNA in low quantities but potentially still sufficient for MinION sequencing (~6 ng/μL), while substitution with lysozyme/OMO/proteinase K treatment enabled consistent recovery of DNA in relatively large quantities (45–72 ng/μL). However, DNA samples obtained using OMO lysis were highly contaminated by protein (1–4 μg/mL), the eluate was white, and the DNA did not migrate toward the positive electrode and remained in the wells of the agarose gel.

Finally, the protocol using multireagent SDS‐based lysis buffer and magnetic beads for recovery (Mayjonade et al., 2016) was inefficient when used as described, but lysis using SDS/proteinase K, OMO/proteinase K, and lysozyme/OMO/proteinase K permitted recovery of sufficiently pure DNA (<200 ng/μL protein) but in low quantity (2–4 ng/μL). However, lysozyme/SDS/proteinase K lysis resulted in good yields of pure DNA (~40 ng/μL) (Table 3). It is noteworthy that these extractions were performed with a contaminants‐removal step (potassium acetate treatment followed by centrifugation) carried out at ambient temperature, unlike the 4°C incubation described in Mayjonade et al. However, when this step was omitted, it resulted in either no DNA recovered (without lysozyme treatment) or low DNA yield (with lysozyme treatment), accompanied by a high amount of contaminating protein (360–560 ng/μL) which prevented DNA migration on the agarose gel.

### 3.2 Refinement of the extraction method

After completing the above experiments, it was established that the extraction method described by Mayjonade et al. (2016) could be efficient when a lysozyme prelysis step is included, and lysis is performed by SDS (1.25%)/proteinase K (0.74 mg/mL) or OMO (1 mg/L)/proteinase K (0.74 mg/mL). A contaminant removal step is critical and cannot be omitted but can be effective at ambient temperature (Table 3). Consequently, these two lysis treatments were evaluated under different culture inputs (1.5, 3, and 4 mL), removing precipitated contaminants by centrifugation (as described) or by syringe filtration as an alternative, centrifuge‐free approach. Extraction from lysozyme/OMO/proteinase K‐treated lysates was only successful with a 4 mL culture. Although the yield was substantial (45‐120 ng/μL), samples contained a high amount (~500 ng/μL) of contaminating protein (Table 4). In contrast, extraction from lysozyme/SDS/proteinase treated lysates was successful in all cases and recovered DNA was sufficiently pure (<200 ng/μL protein) regardless of the method used to remove the precipitated contaminants (centrifugation vs. filtration). The yield increased with an increased volume of culture input. The lowest input (1.5 mL culture) yielded sufficient DNA (~12 ng/μL in 20 μL eluate) for MinION sequencing with the rapid barcoding kit (SQK‐RBK004) on the R9 flow cell. As 400 ng is required for the library, or a minimum of 33 ng per sample for a 12‐sample library, 4.4 ng/μL in 7.5 μL could potentially be sufficient. However, 10–20 ng/μL is preferable as some of the DNA is lost during library preparation.

**Table 4:** Optimization of the method: SDS versus OMO for lysis, centrifuge versus filter for removal of contaminants.

SN	Culture input	Centrifuge/filter paper	Lysis	Concentration of DNA (ng/μL)	Concentration of protein (ng/μL)
1.	1.5 mL	Filter	Lysozyme/SDS/proteinase K	11.73	127
2.		Centrifuge		12.82	115
3.		Filter	Lysozyme/OMO/proteinase K	<0.1	126
4.		Centrifuge		2.34	130
5.	3 mL	Filter	Lysozyme/SDS/proteinase K	20.9	<1.0
6.		Centrifuge		16.2	135
7.		Filter	Lysozyme/OMO/proteinase K	<0.1	<1.0
8.		Centrifuge		1.41	<1.0
9.	4 mL	Filter	Lysozyme/SDS/proteinase K	45.9	201
10.		Centrifuge		99	170
11.		Filter	Lysozyme/OMO/proteinase K	45.0	523
12.		Centrifuge		120	493

*Note*: Lysozyme = 0.16 mg/mL, SDS = 1.25%, OMO = 1 mg/L (w/v), proteinase K = 0.74 mg/mL.

Abbreviations: OMO, OMO detergent laundry powder; SDS, sodium dodecyl sulfate.

**Figure 1:**
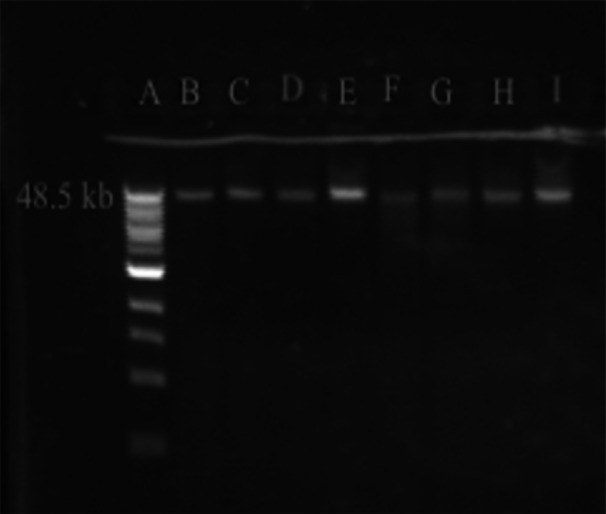
Gel electrophoresis of DNA extracted using the RLS (resource‐limited settings) method. A ‐ Quick‐load 1 kb Extend DNA Ladder (NEB), 48.5 kb upper band. B‐I – duplicate DNA extracts from *Streptococcus iniae* QMA0248 processed by centrifugation/PEG8000 (B‐C), filtration/PEG8000 (D‐E), centrifugation/isopropanol (F‐G), and centrifugation/isopropanol (H‐I).

Subsequent refinement/optimization of the method was performed using lysozyme/SDS/proteinase K treatments applied to 1.5 mL input culture (Table 5). In these experiments, SDS was reduced to 0.5% sufficient for lysis as demonstrated in other methods (Table 3) and combined with proteinase K at 0.3 and 0.5 mg/mL and RNaseA at 0.16, 0.4, and 0.8 mg/mL. Each combination was tested with either centrifugation or filtration to remove precipitated contaminants. Before capture by the magnetic beads, DNA was precipitated using either PEG8000 (Mayjonade et al., 2016) or isopropanol as an alternative approach. It was determined that 0.16 mg/mL RNaseA should be used in the final protocol, as higher concentrations did not decrease contaminating RNA. Proteinase K at 0.3 mg/mL was chosen in the final protocol, demonstrating efficiency comparable to 0.5 mg/mL (Table 5), and in some cases superior to 0.1 mg/mL (data not shown). Across all combinations of the contaminant‐removal methods and DNA precipitation methods (centrifuge/PEG8000; filter/PEG8000; centrifuge/isopropanol; filter/isopropanol), DNA yields and purity were acceptable and consistent, showing efficient and reproducible DNA extraction. HMW DNA was recovered in all cases, evidenced by a single >48 Kb band and negligible smearing on the agarose gel (Figure 1).

**Table 5:** Final refinement of the method: Centrifugation versus filtration for removal of contaminants, PEG8000 versus isopropanol for precipitation of DNA.

SN	Removal of contaminants/DNA precipitation	Lysozyme/SDS	RNase A (mg/mL)	Proteinase K (mg/mL)	DNA (ng/μL)	RNA (ng/μL)	Protein (ng/μL)
1.	Centrifuge/PEG 8000 (Mayjonade et al., 2016)	16 mg/mL/0.5%	0.16	0.3	14	<2.0	<1
2.				0.5	13.7	2.2	<1
3.			0.4	0.3	14.3	2.1	<1
4.				0.5	15.3	2.4	<1
5.			0.8	0.3	16.5	2.55	100
6.				0.5	21.5	2.5	110
7.	Centrifuge/isopropanol	16 mg/mL/0.5%	0.16	0.3	81.8	31.6	134
8.				0.5	60.7	32.4	110
9.			0.4	0.3	76.3	30.1	142
10.				0.5	65.5	28.3	130
11.			0.8	0.3	75.2	30.9	151
12.				0.5	67.9	30.4	145
13.	Filter/PEG8000	16 mg/mL/0.5%	0.16	0.3	28.5	<2.0	<1
14.				0.5	11.1	<2.0	<1
15.			0.4	0.3	11.2	<2.0	<1
16.				0.5	4.12	<2.0	<1
17.			0.8	0.3	11.6	<2.0	<1
18.				0.5	14.7	<2.0	<1
19.	Filter/isopropanol	16 mg/mL/0.5%	0.16	0.3	33.8	21.4	120
20.				0.5	48	18.7	<1
21.			0.4	0.3	28.4	14.7	120
22.				0.5	17.4	32.6	<1
23.			0.8	0.3	58.3	19.2	120
24.				0.5	10.2	14.2	100

Abbreviations: PEG8000, polyethylene glycol 8000; SDS, sodium dodecyl sulfate.

The syringe filtration was as efficient, or even slightly more so, than centrifugation in removing contaminating proteins but somewhat decreased the DNA yields. Isopropanol permitted the recovery of substantially higher quantities of DNA compared to PEG8000 buffer: PEG8000 buffer extracted 4–28 ng/μL with an average of 15 ng/μL, while isopropanol extracted 10–82 ng/μL with an average of 52 ng/μL. Isopropanol‐precipitated DNA samples exhibited similar or slightly higher levels of contaminating protein, proportional to DNA yield, but contained significantly more contaminating RNA.

### 3.3 The final protocol and sequencing of the extracted DNA

Our final optimized DNA extraction method is based on the protocol described by Mayjonade et al. (2016) but uses some major and multiple minor modifications. Reagents and their estimated costs are listed in Appendix Table D1. The protocol is performed step‐by‐step as follows:

(1) Adjust bacterial culture to 10^8^ CFU/mL. Take 1.5 mL culture in a microcentrifuge tube, pellet and discard the supernatant. Add 250 μL TE buffer with 16 mg/mL lysozyme.
*Alternatively (if not using a centrifuge)*: resuspend half of a 10 μL inoculating loop of plate‐grown bacteria in 250 μL TE with 16 mg/mL lysozyme.
(2) Mix thoroughly and incubate at 37°C for 30 min.
(3) Add ~13.5 μL of 10% SDS, ~2.16 μL of 20 mg/mL RNaseA, and ~4.05 μL of 20 mg/mL proteinase K, to obtain 0.5% SDS, 0.16 mg/mL RNaseA, and 0.3 mg/mL proteinase K concentration in a total volume of 270 μL TE buffer.
(4) Mix thoroughly and incubate at 55°C for 30 min.
(5) Add 1/3 volume of 5 M potassium acetate (90 μL).
(6) Mix by pipetting or low‐speed vortex. Incubate at ambient temperature for 10 min.
(7) Centrifuge in a microcentrifuge at >12,000 × *g* for 2 min.
*Alternatively (if not using a centrifuge):* pass suspension through 0.22 µm syringe‐filter.
(8) Add 1 volume (360 μL) of isopropanol or PEG8000 buffer. Mix by pipetting or low‐speed vortex.
(9) Add 20 μL of AMPure XP beads. Mix by pipetting 10–15 times.
(10) Incubate at ambient temperature for 10 min.
(11) Place the tube on a magnetic rack, wait until the beads settle, and discard the supernatant.
(12) Add 250 μL of freshly prepared 80% ethanol and hold for 30 s.
(13) Discard the ethanol, add 250 μL of 80% ethanol, hold for 30 s.
(14) Discard the ethanol. If using a centrifuge, briefly spin, place back on the rack, and remove the residual ethanol.
(15) Remove the tube from the magnetic rack and dry the beads (~1 min if residual ethanol was spun in step 13).
(16) Add 20 μL of nuclease‐free water, and thoroughly resuspend the beads by pipetting 10–15 times.
(17) Incubate at ambient temperature for 10 min.
(18) Place the tube on the magnetic rack, wait until the solution becomes clear, and transfer the eluate into a fresh tube.
(19) Mix by pipetting, quantify (Qubit), and store at −20°C (ideally de‐frost immediately before sequencing; avoid repeated freeze/thaw).

To verify the protocol's suitability for DNA extraction from diverse *Streptococcus* strains and for MinION sequencing, it was tested on five *S. agalactiae* and seven *S. iniae* isolates (Table 2). The centrifuge‐free version of the protocol was applied to *S. iniae*, where cells were collected from agar plates and precipitated contaminants were removed by filtration (steps 1 and 7 in the protocol, respectively). For *S. agalactiae,* the protocol was challenging due to the sticky light growth of sequence type 261 strains on agar plates, making harvest with an inoculation loop difficult. Thus, *S. agalactiae* isolates were grown in broth and pelleted by centrifugation, which was also used to pellet the precipitated contaminants. In both cases, DNA was precipitated using PEG8000. A 12‐sample barcoded library was prepared using the rapid barcoding kit SQK‐RBK004 and sequenced on the Mk1B MinION device using the FLO‐MIN106 (R9.4.1) flow cell.

According to genome assemblies performed with Flye (Kolmogorov et al., 2019), each sample yielded a total of 70–160 million passed reads, with an N50 ranging from 4 to 17 kb and N90 from 1 to 4 kb (with some exceptions, see below). In most cases, one closed chromosome of the expected size (2.1–2.3 Mb for *S. iniae* and 1.8–2.1 Mb for *S. agalactiae*) was generated and confirmed to belong to the respective species through a 16S rDNA homology search using BLASTn (NCBI) (Table 6).

**Table 6:** Sequencing and assembly statistics for *Streptococcus iniae* and *Streptococcus agalactiae* DNA extracted using the final optimized protocol.

Species and strain	DNA (ng/μL)	RNA (ng/μL)	Protein (ng/μL)	Total read length (bp)	Reads N50/N90	Total contig length (bp)	Number of contigs, topology	Contig size and coverage, genome accession
*S. iniae* QMA0084	42.1	3.08	<1	75,793,064	3217/751	2,204,818	8 linear	5251–1,461,907 (mean 37×) JBDPIT000000000
*S. iniae* QMA0139	15.7	<2.0	<1	101,381,072	4114/979	2,370,202	2 circular	2,339,923 (43×) CP159896 **30,279 (70×)** CP159897
*S. iniae* QMA0139_ CTAB^c^	140	<2.0	<1	99,920,467	14,747/3842	2,367,992	2 circular	2,337,784 (38×) CP159894 **30,208 (125×)** CP159895
*S. iniae* QMA0139_ Unicycler^d^	‐	‐	‐	101,381,072 (ONT_RLS) + 99,920,467 (ONT_CTAB) + 552,363,020 (Illumina)	Above+Above+110/110		1 linear 1 circular	2,337,380 (230x) CP158020 **15,146 (309x)** CP158021
*S. iniae* QMA0177	13.7	<2.0	<1	98,379,498	7350/1217	2,110,440	1 circular	2,110,440 (48 x) CP159893
*S. iniae* QMA0186	22.7	<2.0	<1	80,135,092	5770/1423	2,031,215	1 circular	2,031,215 (37×) CP159892
*S. iniae* QMA0249	15.4	<2.0	<1	69,035,516	1467/419	2,140,312	131 linear	2458–94,065 (mean 33×) JBDPIU000000000
*S. iniae* QMA0445	38.2	2.99	<1	71,484,216	4141/910	2,107,970	1 circular	2,107,970 (31×) CP159891
*S. iniae* QMA0462	97.2	8.88	<1	528,352,428	16,989/4222	2,128,764	1 circular	2,128,764 (251×) CP159948
*S. agalactiae* QMA0274	13.9	<2.0	<1	164,579,362	14,434/3737	1,839,058	1 circular	1,839,058 (91×) CP159890
*S. agalactiae* QMA0321	24.6	<2.0	<1	41,051,780	17,751/2667	3,969,592	2 circular	2,130,949^a^ (6×) 1,838,643 (15×) CP159889
*S. agalactiae* QMA0496	23.4	<2.0	240	27,154,429	14,283/2676	1,848,470	1 circular	1,848,470 (15×) CP158377
*S. agalactiae* QMA0522	47	<2.0	200	109,225,544	17,978/4437	4,275,044	3 circular	2,385,942^b^ (26×) 50,510^b^ (86×) 1 838 592 (22×) CP158376
*S. agalactiae* QMA0539	51.4	<2.0	200	157,817,495	11,648/2822	2,083,884	1 circular	2,083,884 (77×) CP157196

*Note*: Bold font – *S. iniae* plasmid pSI1 (dimer of 30 kb, a misassembly, or a monomer of 15 kb, true size).

^a^ Contaminating DNA (*S. iniae*).

^b^ Contaminating DNA (*Staphylococcus capitis*).

^c^ DNA extraction performed using lysozyme + CTAB (reference method).

^d^ Hybrid genome assembly (Unicycler).

The exceptions were with the reads from *S. iniae* QMA0084 and QMA0249 which were only assembled into 8 and 131 linear contigs, respectively, due to the high amount of DNA fragmentation in these samples, as indicated by the low N50/N90 values (3/0.7 and 1.5/0.4 kb). This fragmentation may be attributed to higher nuclease content in these strains or nuclease contamination during handling, rather than an issue with the extraction method, considering much higher N50/N90 in other samples' reads. Nonetheless, the total contig length in these draft assemblies suggests the full representation of the genome. In addition, two assemblies were derived from mixed broth cultures, a common problem with field isolations and propagation of slow‐growing fastidious Streptococci from ST261 serotype 1b. The assembly expected from *S. agalactiae* QMA0321 contained two complete circular chromosomes, one of 1.84 Mb identified as *S. agalactiae* and a second chromosome of 2.13 Mb identified as contaminating *S. iniae* sequence (Table 6). The assembly expected from *S. agalactiae* QMA0522 contained three circular contigs, one chromosome of 1.84 Mb identified as *S. agalactiae*, a second chromosome of 2.39 Mb identified as *Staphylococcus capitis* contaminant, and a 50 kb *S. capitis* plasmid. However, a circular contig of ~30 kB was also found in the assembly of *S. iniae* QMA0139, a preparation free from contaminating DNA (Appendix Figure A1a). This element was investigated as a putative uncharacterized *S. iniae* plasmid (see below).

### 3.4 Characterization of pSI1 – A 15 kb low‐copy number mobilizable plasmid of *S. iniae*

To confirm the presence of plasmid in *S. iniae* QMA0139, its genome sequencing and assembly were repeated using DNA extracted by the lysozyme + CTAB (reference) method. As in the case of DNA extracted using RLS protocol, a ~30 kB circular contig was present in the assembly (Table 6; Appendix Figure A1b). High sequence homology (>97% identity, >70% query cover with BLASTn) to plasmids pC10B, pl11C, and p267B from *Lactococcus lactis* (CP034582.1; CP069226.2; CP032060.2) and an unnamed plasmid from *Streptococcus dysgalactiae* (CP116873.1) supported that the 30 kb contig represents a plasmid sequence. A ~twofold and ~threefold increase in coverage compared to the chromosome coverage in the “RLS‐assembly” and the “CTAB‐assembly,” respectively, indicated a low copy number of the plasmid. There are, to date, no published records of *S. iniae* plasmids that we are aware of, and two plasmid entries of 14 and 17 kb in the NCBI database (CP024844.1 and CP125109.1, respectively) are unnamed. Hence, we named this plasmid pSI1.

**Figure 2:**
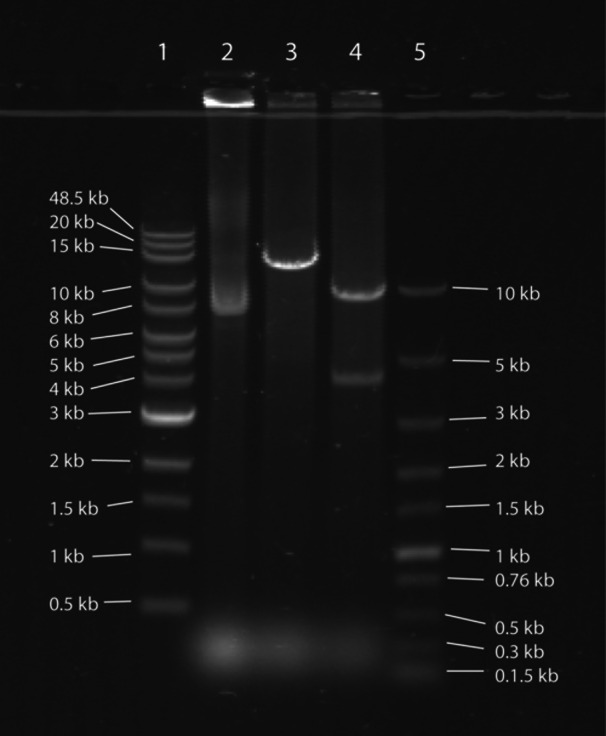
pSI1 plasmid extracted from *Streptococcus iniae* QMA0139, visualized 0.5% TAE agarose gel. Lane 1 – Quick‐Load 1 kb Extend DNA Ladder (NEB); Lane 2 – 1 μg of undigested pSI1; Lane 3 ‐ 1 μg of pSI1 digested with NsiI (NEB); Lane 3 ‐ 1 μg of pSI1 digested with AfIII (NEB); Lane 4 – Fast DNA Ladder (NEB).

**Figure 3:**
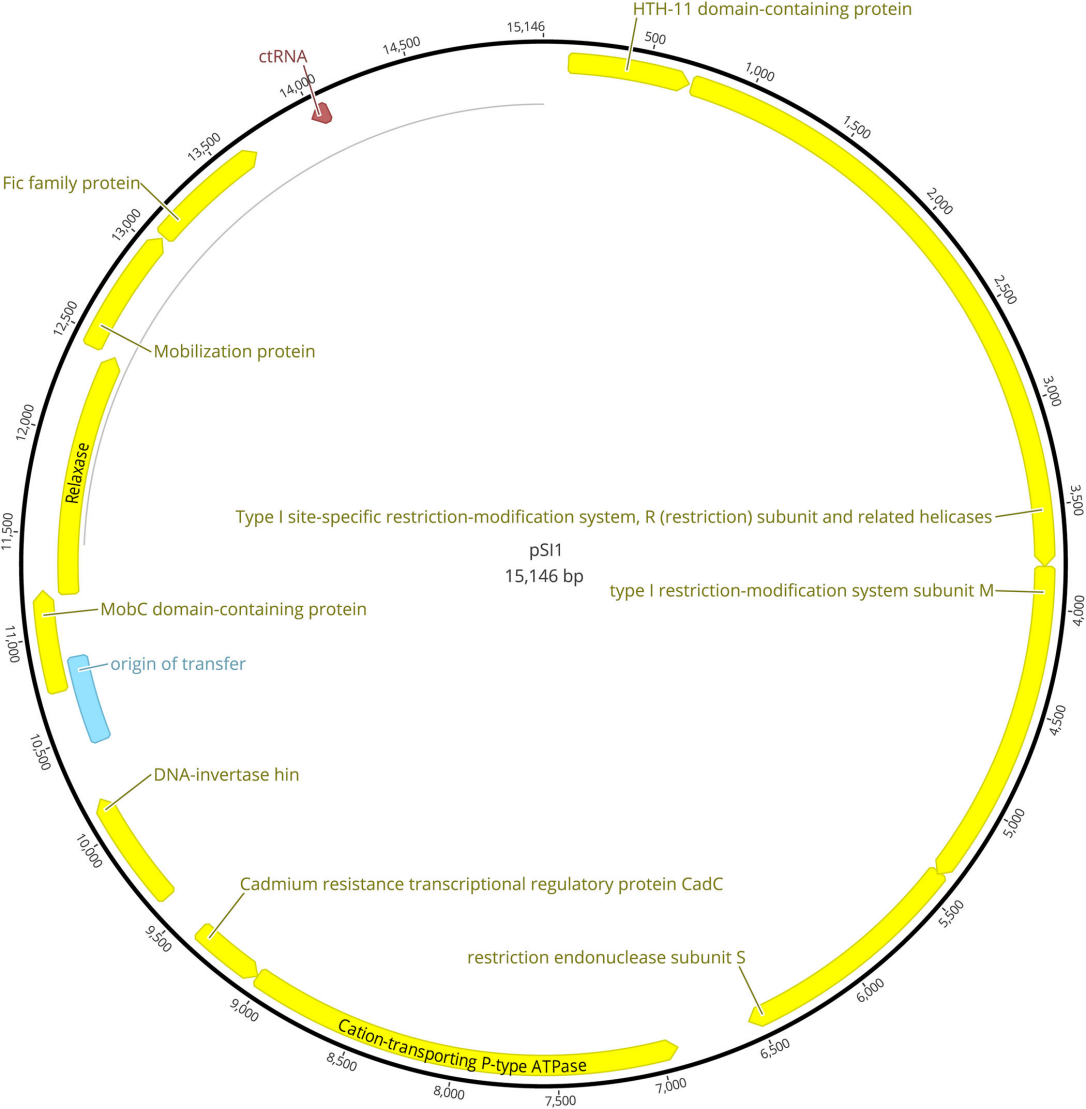
pSI1 plasmid contig (CP158021) from genome assembly of *Streptococcus iniae* QMA0139 generated by Unicycler, annotated with Bakta, and visualized in Geneious Prime.

It was evident from gene annotations (PROKKA) that 30 kb pSI1 contig is a dimeric sequence (Appendix Figure B1), which was further confirmed by the prediction of restriction cutting sites (no 1‐cutters; only 2‐cutters at mirror sites; Appendix Figure C1). To determine whether the dimeric sequence was a misassembly or occurred naturally, pSI1 was extracted and visualized (Figure 2). Migration of the pSI1 digested with NsiI and AfIII enzymes confirmed the predicted dimer cutting sites for 30 kb: 2 (mirror) cuts by NsiI resulting in two monomers of 15 kb (Figure 2, Lane 3), and 4 cuts by AfIII resulting in 10 and 5 kb bands seen on the gel (Figure 2, Lane 4). Yet, the same digestion products would be seen for the 15 kb plasmid monomer (1 cut for Nsi, which would linearize the monomer, and 2 cuts for AfIII), and migration of the undigested sample down to 8 kb (Figure 2, Lane 2) is more likely to occur for 15 kb supercoiled monomer than for 30 kb supercoiled dimer. Indeed, multiple (*n* = 22) reads of ~15 kb were mapped to the 30 kb dimer assembly, but no reads of a longer size were mapped. Thus, both migration of the undigested pSI1 and read mapping suggested that pSI1 is a monomer of ~15 kb and that the 30 kb dimeric sequence is an assembly artifact. Hybrid assembly using a short‐read‐first algorithm is recommended to address the current issue of doubling or tripling the size of small plasmids (<20 kb) by long‐read assemblers (Bouras et al., 2023; Johnson et al., 2023; Wick & Holt, 2019). Hence, a Unicycler assembly using QMA0139 Illumina reads from a previous study (SRR7151914) and long reads from this study were carried out. Although this hybrid assembler failed to circularize the chromosomal contig (Appendix Figure A1c), pSI1 was indeed recovered as a closed contig of 15,146 bp (CP158021) (Figure 3; Appendix Figure A1c). pSI1 comprises 13 sequence annotations identified by Bakta (Schwengers et al., 2021): 4 genes encoding type 1 restriction‐modification system enzymes, *cadA* and *cadC* encoding cation‐transporting P‐type ATPase and cadmium resistance transcriptional regulator respectively, *hin* encoding DNA‐invertase, two genes encoding Mob proteins, one gene encoding relaxase, one gene encoding Fic protein, an origin of transfer sequence OriT, and ctRNA (Figure 3). Thus, pSI1 is a mobilizable plasmid, and its copy number is controlled by ctRNA, which functions as a rolling‐circle replication inhibitor (Venkova‐Canova et al., 2003).

## 4 DISCUSSION

In this study, we investigated various methods for extraction of HMW DNA from *Streptococcus* spp. for MinION sequencing, with a focus on their applicability in RLS, such as field conditions and/or LMIC. In the first instance, five published DNA extraction methods were evaluated, either as originally described or with RLS‐appropriate modifications, to assess their ability to yield DNA of sufficient quantity and quality for PCR‐free rapid library preparation ONT kits (e.g., SQK‐RBK004, SQK‐RBK114.24). The effective extraction method was further optimized and refined, then applied to diverse strains of *S. iniae* and *S. agalactiae*, and verified by sequencing.

Successful extraction of genomic DNA from bacteria requires the complete degradation of the peptidoglycan layer and cell membrane (lysis), followed by purification (separation, recovery) of the DNA from other components (Barbosa et al., 2016). Gram‐positive bacteria have a thick peptidoglycan layer (cell wall), making them recalcitrant to lysis and generally require treatments with peptidoglycan‐weakening/disrupting treatments, such as mechanical disruption or digestion with lysozyme, mutanolysin, and/or lysostaphin (Coleman et al., 1970; Gill et al., 2016; Şahin et al., 2016). Consistent with this, the DNA extraction protocol (Wilson, 2001) routinely employed in our laboratory for MinION sequencing of DNA from gram‐negative bacteria (Baseggio et al., 2022) was only efficient for the extraction of streptococcal DNA after the inclusion of the lysozyme treatment. Lysozyme hydrolyzes β(1‐4)‐linkages between N‐acetylmuramic acid and N‐acetyl‐D‐glucosamine residues in peptidoglycan (Ferraboschi et al., 2021). However, although the method is inexpensive and very effective for the recovery of highly pure HMW DNA, it relies on toxic and carcinogenic chemicals – CTAB for the removal of contaminants and phenol and chloroform for DNA recovery. Consequently, it is unsuitable for use in locations where there are limited means of personal protection measures (e.g., no fume hood) and proper waste disposal systems (Ali et al., 2017). Nonetheless, the CTAB method of Wilson (2001) modified to incorporate a lysozyme treatment is a reliable way of streptococcal DNA extraction for nanopore sequencing in standard laboratory settings (Pinzauti et al., 2022). In this study, it provided a reference against which results of extractions using RLS‐suitable methods were compared.

Lysis using laundry powder and DNA recovery from lysates via capture on cellulose dipstick are simple, nonhazardous approaches which offer very attractive options for DNA extraction in RLS. Laundry powder contains a combination of detergents and enzymes (proteases, lipases), and its effectiveness in DNA extraction was reported in animal and plant cells (Bahl, 1996) and from the gram‐negative bacterium *Pseudomonas aeruginosa* (Mirnejad et al., 2012). In the case of *P. aeruginosa,* 402 ng/μL of DNA with purity and quality better than the manual phenol‐chloroform extraction method was extracted using laundry powder at 40 mg/L (Mirnejad et al., 2012). However, this protocol was inefficient in DNA extraction from *S. iniae* when used as described (laundry powder lysis/ethanol recovery), even at much higher concentration of the laundry powder up to saturation (60–1000 mg/mL OMO powder) combined with lysozyme and proteinase K treatments. DNA recovery using ethanol was also unsuccessful when applied to lysozyme/SDS/proteinase K lysates (reference lysis method). Likewise, although the DNA of gram‐negative *Campylobacter spp*. captured (from proteinase K lysates) using a cellulose dipstick was successfully used for PCR amplification, the authors indicated that the dipstick does not substantially concentrate DNA in a sample (Mason & Botella, 2020). Indeed, no detectable amounts of streptococcal DNA were extracted using cellulose dipstick from either lysozyme/OMO/proteinase K lysates or lysozyme/SDS/proteinase K lysates.

Magnetic separation is a simple, nonhazardous, and highly efficient way for DNA recovery compatible with RLS applications, where negatively charged DNA molecules are bound to the positively charged silica‐coated magnetic beads, which are then collected on a magnet, allowing proteins and contaminants to be removed by washing (Oberacker, Stepper, Bond, Höhn, et al., 2019). Commercially available magnetic racks are unreasonably expensive, but they can be cheaply and easily assembled in‐house; magnetic beads can also be synthesized at a low cost (Oberacker, Stepper, Bond, Höhn, et al., 2019; Oberacker, Stepper, Bond, Hipp, et al., 2019). However, commercial magnetic beads could be time‐saving and still affordable for LMICs if used in small volumes, such as 20 μL AMPure XP per sample used in extractions here. The first magnetic separation method we tried was Bio‐On‐Magnetic‐Beads (BOMB) gDNA extraction using GITC lysis, following protocol #7.1 from the BOMB platform (Oberacker, Stepper, Bond, Hipp, et al., 2019). The lysis buffer in this protocol employs multiple reagents, including toxic GITC, and is not suitable for RLS. Therefore, we replaced the GITC‐based lysis with lysozyme/SDS/proteinase K and lysozyme/OMO/proteinase K treatments. However, these modifications resulted in either low DNA yield or good DNA yield but with significant protein contamination, respectively.

Therefore, we evaluated another magnetic bead‐based extraction protocol which uses a multireagent SDS‐containing buffer for lysis (Mayjonade et al., 2016). The latter method was developed for HMW DNA extraction for long‐read sequencing applications and yielded 54 ng/μL of 50–100 kb DNA from gram‐negative *Escherichia coli*, which was verified by sequencing on the PacBio platform. Importantly, the removal of protein and polysaccharide contaminants is carried out between lysis and DNA capture on the beads. For this, lysates are incubated with potassium acetate at 4°C which leads to the formation/precipitation of insoluble potassium dodecyl sulfate complexes with proteins and polysaccharides, which are subsequently removed by centrifugation. Initially, we carried out the extraction as described except for SDS/proteinase K and lysozyme/OMO/proteinase K for lysis (with and without prior lysozyme treatment) and with potassium acetate treatment omitted or performed at ambient temperature. Lysis using lysozyme/SDS/proteinase K treatment was highly efficient, resulting in a good yield of DNA (54 ng/μL), while lysis using SDS/proteinase K, OMO/proteinase K, or lysozyme/SDS/proteinase K treatments provided low yields of DNA (2.5–4.5 ng/μL). These lysis treatments are more convenient/efficient compared to the lysis approach described in Mayjonade et al. employing a six‐component buffer which needs to be prepared fresh for each extraction and incubated at 65°C for at least 30 min. Not surprisingly, samples not treated with potassium acetate were heavily contaminated, as indicated by excessive protein content (>250 ng/μL protein) and did not move out of the wells of an agarose gel, confirming that this step is critical for the purity of the recovered DNA. In contrast, incubation of the lysates with potassium acetate performed at ambient temperature (instead of 4°) yielded sufficiently pure DNA samples (>250 ng/μL protein; samples migrating on the gel) and thus represents a convenient modification for RLS. In addition, washing of the magnetic beads suggested by the original protocol proved to be unnecessary and was omitted, which further simplified the extraction procedure.

In the second extraction, we applied lysozyme/SDS/proteinase K and lysozyme/OMO/proteinase K lysis to three different inputs of streptococcal culture. Removal of contaminants precipitated by potassium acetate (at ambient temperature) was carried out either by centrifugation (Mayjonade et al., 2016) or by syringe filtration – a convenient modification allowing DNA extraction without the use of an electrical power supply. Lysozyme/SDS/proteinase K lysis yielded 12–13, 16–21, and 46–99 ng/μL of sufficiently pure DNA from 1.5, 3, and 4 mL streptococcal culture, respectively – DNA recovery proportional to the culture input, which indicates effective reproducible lysis combined with successful removal of contaminants. Samples that were filtered for removal of contaminants were as clean as, or cleaner than, centrifuged samples, indicating the success of the modification. Lysozyme/OMO/proteinase K lysis yielded none or very small amounts of DNA (1.5–2.4 ng/μL) from 1.5 and 3 mL cultures, but 45–120 ng/μL of it was recovered from 4 mL culture. Although the latter yields were equal to yields from 4 mL culture treated with lysozyme/SDS/proteinase K, these samples had high concentrations of contaminating protein. Most likely, in contrast to SDS, OMO powder did not provide the dodecyl sulfate groups required for the formation of insoluble potassium dodecyl sulfate complexes with proteins and polysaccharides during incubation with potassium acetate.

Finally, we optimized the method using lysozyme/SDS/proteinase K lysis to minimize the use of reagents and time and to successfully extract DNA from *Streptococcus* spp. The procedure takes approximately 1 h 30 min, including 30 min of lysozyme treatment (which may be omitted for gram‐negative bacteria). We estimated the total cost of the extraction per sample to be around AU$1.25 if molecular biology reagents are purchased from reputable suppliers. More than 2/3 of the total cost comes from the price of the AMPure XP bead and lysozyme, estimated at AU$0.50 each. Therefore, in‐house synthesis of the magnetic beads (Oberacker, Stepper, Bond, Hipp, et al., 2019) and longer incubation with lower concentration of lysozyme may be considered for RLS, where minimization of the cost should be prioritized over convenience. We found that significantly higher yields can be recovered if DNA is precipitated (before its capture by the magnetic beads) using isopropanol in the place of the PEG8000 buffer proposed by the original protocol. This result was true regardless of whether protein/polysaccharide complexes with potassium dodecyl sulfate were removed by centrifugation or filtration. It was noted by the original authors that PEG8000 precipitation yields purer DNA compared to isopropanol precipitation. This was not supported by our results for protein contamination but was true for RNA contamination. Thus, isopropanol, which may be more widely available in RLS/LMIC, appears to be more efficient than PEG8000 in precipitation of both nucleic acids leading to samples with higher DNA yields but also higher RNA contamination.

The final step‐by‐step extraction protocol based on the method of Mayjonade et al. (2016) contains multiple minor and some major modifications (e.g., lysozyme treatment, simplified lysis, potassium acetate treatment at ambient temperature, removal of precipitated contaminants by filtration) and can be found in Section 3.3. Its suitability for diverse streptococcal strains was ascertained by successful DNA extraction and MinION sequencing of seven *S. iniae* and five *S. agalactiae* isolates selected from phylogenetically phenotypically distinct groups. The extracted DNA ranged from 13 to 97 ng/μL (260–1940 ng total yield) and, with minor exceptions, had a HMW of ~50 kB and yielded long reads with N50 of 4–17 kb and N90 of 1–4 kb (fragmentation‐based library; SQK‐RBK004 kit), which were assembled into complete circular chromosomes ascertaining the robustness of the RLS method described here. Identification of the disease‐causative agent can be performed directly from raw Nanopore reads within hours of the sequencing run using EPI2ME What's In My Pot (WIMP) Workflow (ONT), even with sequencing on high error rate (legacy) R9 flow cells used in this study.

With the release of R10 flow cells and base calling accuracy continually improving, robust variant detection from ONT reads has become feasible (Ni et al., 2023), that is, identification of MLST, serotype, and antibiotic resistance mutations of streptococcal isolates is possible (but subject to database availability). In addition, nanopore sequencing can resolve contaminating DNA, which is critical for diagnostics of disease from isolations performed in the field/DNA extracted in the field. Here, it was demonstrated by the resolution of contaminants introduced during the inexpert broth culture of two slow‐growing fish‐adapted ST261 *S. agalactiae* isolates, which are easily overgrown by less fastidious environmental bacteria. The utility of long‐read sequencing was further illustrated by our discovery of a 15 kb low copy number mobilizable plasmid in *S. iniae* QMA0139 (Irion et al., 2021; Rudenko et al., 2020), homologous to plasmids found in other *Streptococcus* spp. and *Lactococcus* spp. Notably, pSI1 was initially misassembled as a plasmid dimer, which is a common occurrence with current long‐read assemblers (Bouras et al., 2023; Johnson et al., 2023; Wick & Holt, 2019). To reflect that this is the first published record of plasmids in *S. iniae*, we named it pSI1.

## 5 CONCLUSION

MinION is an affordable and portable device suitable for whole genome sequencing in RLS, making it ideal for diagnostics and control of *S. iniae* and *S. agalactiae* disease outbreaks in LMIC finfish aquaculture. However, this application requires a DNA extraction method that can recover HMW DNA from species of gram‐positive bacteria that are recalcitrant to lysis and is appropriate for use in RLS/LMIC. This means the method must use nonhazardous, cheap reagents and equipment. In this study, we established an efficient and simple protocol that satisfies these criteria and verified its suitability for rapid library MinION sequencing of diverse fish‐pathogenic streptococcal strains. The value of HMW DNA extraction coupled with long‐read sequencing was demonstrated by the recovery of a novel plasmid in one of the *S. iniae* genome assemblies. We named this plasmid pSI1 to reflect that it is the first published report of natural plasmids in this species*.*

## AUTHOR CONTRIBUTIONS

**Suvra Das**: Investigation; writing—original draft; validation; visualization; software. **JérÔme Delamare‐Deboutteville**: Writing—review and editing; funding acquisition; supervision; methodology; conceptualization. **Andrew C. Barnes**: Writing—review and editing; conceptualization; funding acquisition; methodology; supervision. **Oleksandra Rudenko**: Conceptualization; writing—original draft; methodology; visualization; writing—review and editing; supervision; investigation; validation; software.

## CONFLICT OF INTEREST STATEMENT

The authors declare no conflict of interest.

## ETHICS STATEMENT

None required.

## Data Availability

Genome assemblies and associated reads from this study were deposited at NCBI Genomes and SRA, respectively, under the NCBI BioProject PRJNA1082471: https://www.ncbi.nlm.nih.gov/bioproject/PRJNA1082471.

## APPENDIX D

**Table D1:** Reagents used in the final DNA extraction protocol and their estimated cost per sample.

Reagent	Supplier	Quantity and cost^a^	Amount used per sample	Cost per sample^b^
Lysozyme from chicken egg white	Sigma‐Aldrich (L6876)	1G, $117.00	0.004 g (for 250 μL of 16 mg/mL solution in TE buffer)	$0.47
TE Buffer, 100X	Sigma‐Aldrich (574793)	100X, 1 L $348.10	250 μL of 1X	$0.01
SDS	Sigma‐Aldrich (L3771)	25 g, $80.10	0.00135 g (for 13.5 μL of 10% solution)	$0.01
RNaseA (20 mg/mL)	Thermo Fisher Scientific (12091021)	10 mL, $281.00	2.16 μL	$0.06
Proteinase K (20 mg/mL)	Thermo Fisher Scientific (25530049)	5 mL, $286.00	4.05 μL	$0.23
Potassium acetate	Sigma‐Aldrich (529543)	250 g, $122.00	0.044 g (for 90 μL of 5 M solution)	$0.02
Polyethylene glycol 8000 (PEG8000)	Thermo Fisher Scientific (043443.36)	500 g, $136.39	0.07 g (for 360 μL of PEG8000 buffer)	$0.02
Sodium chloride, BioUltra, for molecular biology, ≥99.5% (AT)	Sigma‐Aldrich (71376)	1 kg, $211.00	0.06 g (for 360 μL PEG8000 buffer)	$0.01
Isopropanol, for molecular biology, BioReagent, ≥99.5%	Sigma‐Aldrich (I9516)	500 mL, $109	360 μL	$0.08
AMPure XP Bead‐Based Reagent	Beckman Coulter (A63881)	60 mL, $1457	20 μL	$0.48
Ethanol, 200 proof for molecular biology	Sigma‐Aldrich (E7023)	500 mL, $144.00	0.4 mL (for 0.5 mL of 80% solution)	$0.11
Nuclease‐Free Water	Qiagen (129117)	5 L, $157	123.5 μL if isopropanol used (13.5 μL for SDS solution; 90 μL for potassium acetate solution; 20 μL for DNA elution) Or 483.5 μL if PEG8000 buffer is used (+360 μL for PEG8000 buffer)	S0.01
Total if PEG8000 buffer is used				$ 1.24
Total if isopropanol is used				$ 1.29

^a^ Retail prices in AUD ex tax.

^b^ Rounded up to the nearest AU$0.01.
